# Polymer Replicas of Fs-Laser-Induced Periodic Surface Structures for Cell Attachment

**DOI:** 10.3390/ma19061091

**Published:** 2026-03-12

**Authors:** Prunella Ndjogo, Marion Widhalm, Agnes Weth, Sebastian Lifka, Werner Baumgartner, Yoan Di Maio, Johannes Heitz

**Affiliations:** 1 Manutech-USD, 20 Rue Pr. Benoit Lauras, 42000 Saint-Etienne, France; prunella.ndjogo@manutech-usd.fr (P.N.); yoan.di-maio@manutech-usd.fr (Y.D.M.); 2 Institute of Applied Physics, Johannes Kepler University Linz, Altenberger Strasse 69, 4040 Linz, Austria; marion.widhalm@jku.at; 3 Institute of Biomedical Mechatronics, Johannes Kepler University Linz, Altenberger Strasse 69, 4040 Linz, Austria; agnes.weth@jku.at (A.W.); sebastian.lifka@jku.at (S.L.); werner.baumgartner@jku.at (W.B.)

**Keywords:** laser-induced periodic surface structures (LIPSS), ultra-fast laser processing, polished titanium samples, polished steel samples, polymer replicas, medical implants, Schwann cells, fibroblasts

## Abstract

We describe the formation of LIPSS by fs laser irradiation on polished titanium or steel samples, from which polymer replicas can be produced. The irradiation of inclined samples allows a variation in the periodicity of the LIPSS in a range between about 500 and 1000 nm, depending on the angle of incidence and the orientation of the laser polarization relative to the plane of incidence, either parallel (p-polarization) or perpendicular (s-polarization). For p-polarization, a second larger-size LIPSS feature with periodicities between about 1300 and 2200 nm is observed at medium angles. LIPSS lines are oriented perpendicular to the light polarization, except for s-polarization on steel samples, where a rotation of up to 35° is observed. In a two-step process, the LIPSS are replicated in polymers. We investigate the attachment of Schwann cells and fibroblasts seeded thereon, which show no direct dependence on the variation in the LIPSS periodicities.

## 1. Introduction

One strategy for treating large nerve injuries is the use of synthetic neural implants made from polymeric materials. These devices are typically hollow, tubular conduits that bridge the two severed nerve ends, aiming to promote the growth of newly formed axons [1]. In this setting, neurons are supported and protected by glial cells, with Schwann cells being particularly important. Schwann cells produce myelin sheaths that insulate and safeguard axons [2] and are essential for axon regeneration after injury [3]. A crucial factor is the stimulation Schwann cells receive from their surrounding environment—either the native extracellular matrix (ECM) or, in artificial implants, the supporting material composed of synthetic polymers or biopolymers—which can influence their elongated alignment and, in turn, the regeneration of axons [3,4].

Recently, we investigated polycarbonate (PC) and cellulose acetate butyrate (CAB) under these aspects [5]. The latter is a biocompatible, biodegradable polymer whose primary component is the biomaterial cellulose, bearing acetyl and acyl groups. It is used as an artificial silk and can be produced in fiber form. CAB has a good potential for medical implants [6]. Laser-induced micro- and nanostructures on PC and CAB surfaces, generated by irradiation with a linearly polarized 1040 nm femtosecond laser, were employed to direct Schwann cell growth. Schwann cells can be guided to grow along a defined direction determined by the orientation of micro-grooves, whereas cells cultured on unstructured surfaces exhibit omnidirectional growth. For PC, high-spatial-frequency laser-induced periodic surface structures (HSF-LIPSS; see, e.g., [7]) with periodicities of a few hundred nanometers on the walls of the micro-grooves provided an additional orienting effect by guiding Schwann cell protrusions. However, the alignment was not complete, likely because the LIPSS on PC were too small in terms of structure height and/or periodicity to achieve full alignment. At CAB, no LIPSS could be produced in this previous study.

LIPSS are increasingly considered for applications in medical materials [8,9,10,11]. However, the formation of LIPSS on polymeric materials, like CAB and PC, by direct laser irradiation can be challenging or result in insufficient parameter ranges for certain applications, as is outlined above. An alternative is a process where the LIPSS are formed on metal samples, which can be used as a mold for replication in polymeric materials [12,13]. A variation in the LIPSS periodicity and height is possible by irradiation of inclined samples [14,15,16,17,18,19], typically with an either parallel (p-polarization) or perpendicular (s-polarization) orientation of the laser polarization relative to the plane of incidence. Depending on the material of the samples, the angle of incidence, and the orientation of the polarization, different periodicities for the LIPSS are observed. Mostly, the orientation of the lines of the LIPSS are perpendicular to the polarization of the light (defined by the direction of the electrical field, ***E***). But under certain conditions, a rotation of the LIPSS lines relative to the normal of the polarization direction is reported.

In the present study, we systematically investigate the dependence of 1040 nm fs-second-laser-induced LIPSS on tilted polished titanium and steel samples on the irradiation parameters. These samples are used as molds for a two-step fabrication of positive replicas in polymer materials. Schwann cells and fibroblasts are seeded on polymers with replicated LIPSS and their attachment is evaluated by means of scanning electron microscope (SEM) images of the cells.

## 2. Materials and Methods

### 2.1. Metal Samples

The titanium alloy Ti-6Al-4V ELI (Extra Low Interstitial) is a frequently used material for medical implants. Square plates consisting of this alloy (20 mm × 20 mm) were provided by Zapp Precision Metals GmbH (Schwerte, Germany). Additionally, steel samples (i.e., consisting of the austenitic Cr-Ni stainless steel 1.4301) with the same dimensions were used. The composition of the specific titanium alloy and steel grade are listed in Table S1, based on the datasheets [20,21]. The samples were polished using a grinding machine (MetaServ, Buehler, Coventry, UK). Sequential grinding was carried out with SiC papers K320, K400, K600, K1200, and K4000 (Hermes; supplied by A.B. Technics, Tribuswinkel, Austria) for 15–20 min, 8 min, 8 min, 5 min, and 5 min, respectively, at 200 rpm and 22 lbf. This was followed by polishing with a cloth with diamond suspensions (Schmitz Metallographie GmbH, Herzogenrath, Germany) of 6 µm, 1 µm, and 0.05 µm particle size for 30 min, 20 min, and 20 min, respectively, at 150 rpm and 20 lbf.

### 2.2. Laser Structuring

Ultra-short-pulse (fs) laser processing enables the creation of very fine surface features because surface melting is often avoided. In this study, laser processing at the Institute of Applied Physics in Linz was performed using an Ytterbium-based amplified femtosecond laser (Spirit 1040-16 HE, Spectra-Physics, Rankweil, Austria) with wavelength  *λ* = 1040 nm , pulse duration  *τ* = 300 fs, typical average power *p* ≈ 5 to 50 mW , and a typical repetition rate  *f* = 0.5 kHz achieved by pulse picking from the 200 kHz fundamental frequency. The linearly polarized beam was focused onto the sample with a 100 mm focal-length lens (LB1676-B-ML, Thorlabs, Bergkirchen, Germany), yielding a Gaussian beam profile with a diameter  2*w*_0_ = 63.4 μm as determined by Liu’s method [22,23]. The power *p* was measured before the lens using a power meter (TPM-A.1, Gentec, Olching, Germany). In calculating the pulse energy *E* and peak fluence *F*_0_, lens transmission losses of about 6.7% were taken into account. Two linear positioning stages enabled lateral sample motion in the *x*- and *y*-directions during irradiation. More details on the setup and the formulas for calculations of the peak fluence, *F*_0_, and the number of laser pulses per area, *N*_eff2D_, are described in our previous work [5,24]. Comparison experiments were performed at Manutech-USD in St. Etienne using a 1030 nm diode-pumped Yb:YAG laser (Tangerine, Amplitude Laser, Pessac, France) with a pulse length of 240 fs and a repetition rate of 1 kHz.

**Figure 1:**
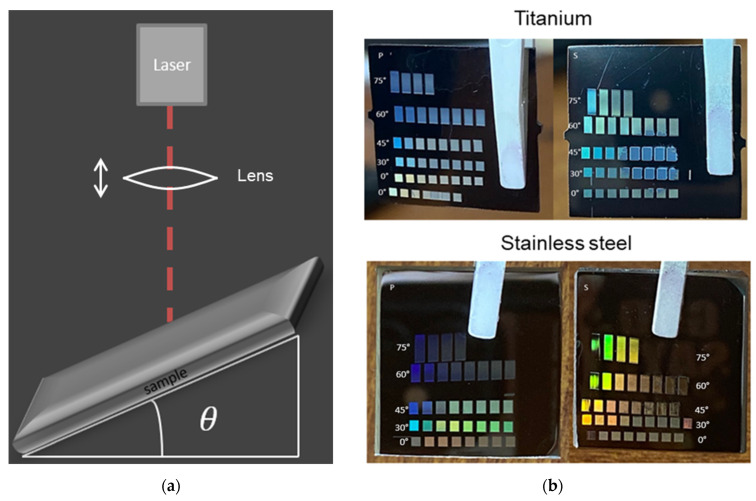
(**a**) Schematic of the irradiation geometry; (**b**) Exemplary photographs of laser-structured polished titanium alloy and steel samples, held by white tweezers, with different irradiation angles and laser fluences at each irradiated area. The white arrow in (**a**) indicates that the position of the lens is adjustable.

The schematic of the laser irradiation geometry is depicted in Figure 1a. The polished samples were arranged on the xy-stages in an inclined position under the inclination angle, *θ*, by means of wedges with defined angles. The different height with variation in the angle was compensated by adjusting the position of the focusing lens, which was placed between the laser and the sample surface. The lens was fixed in the vertical direction on a manual translation stage and was moved manually to adjust the focal distance between the lens and the sample for each angle. This distance was varied until one could observe the brightest plasma at the sample surface. The xy-stages typically scanned an area of 1 mm × 1 mm. For inclined angles, this resulted in rectangular spots on the sample with one side longer than the other (i.e., 1 mm × (1/cos*θ*) mm), as is shown in Figure 1b. The incident laser power was increased accordingly, to obtain the same fluence as for non-inclined areas. The inclination was done in such way that the (fixed) linear laser light polarization was either parallel (p-polarization) or perpendicular (s-polarization) to the plane of incidence. As can be seen in Figure 1b, the irradiated spots often show iridescence with a color depending on the angle of the (white-light) illumination during observation.

### 2.3. LIPSS Measurement Methods

**Figure 2:**
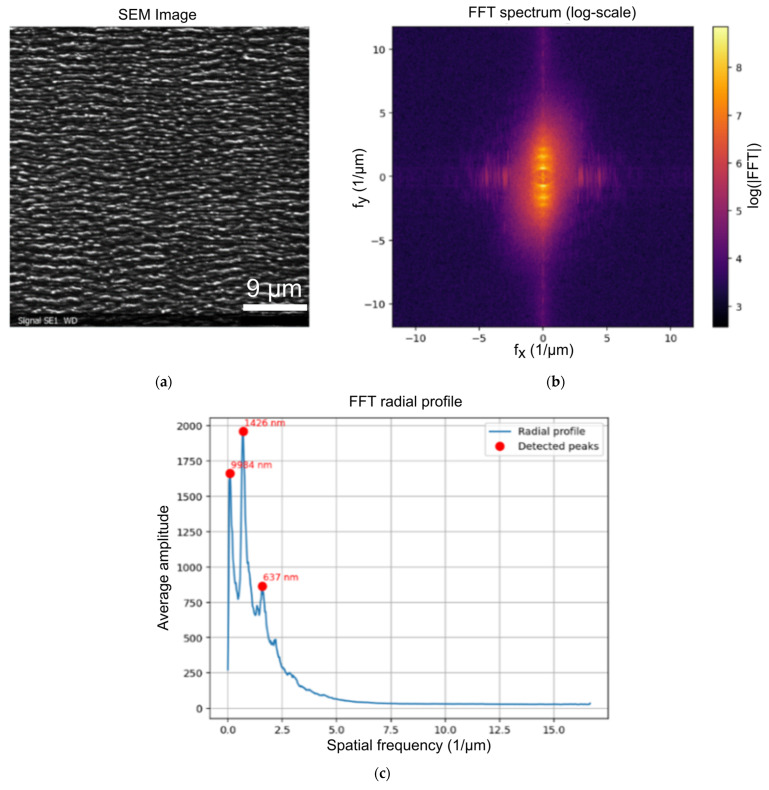
Typical images relevant for the Fourier analysis: (**a**) SEM image of LIPSS pattern; (**b**) 2D FFT image with logarithmic scale; (**c**) radial profile with meaningful peaks at 1426 nm and 637 nm.

The laser-irradiated samples were first examined via scanning electron microscopy (Philips SEM 525 M, Philips GmbH, Kassel, Germany). The periodicity of the LIPSS pattern was then determined by means of Fourier analysis. Figure 2b,c show images of the Fast Fourier Transformation (FFT) for LIPSS generated by p-polarized laser light at an incident beam angle of *θ* = 30° and a fluence of *F*_0_ = 0.55 J/cm^2^ (Figure 2a). This method is used to automatically detect the peaks in SEM images. We used a Python (version 3.13) code to extract the two-dimensional (2D) FFT, which allowed us to determine the LIPSS spatial periodicity. The purpose of this code is to determine the characteristic periodicities of surface structures from an SEM image using Fourier analysis. In the Supplementary Materials, we have included the original Python code as Table S2. First, the image is normalized and converted into the frequency domain through the 2D FFT. The frequency spectrum is then smoothed and averaged radially to obtain a one-dimensional profile that highlights the dominant spatial frequencies. Peaks in this radial profile are automatically detected, and their corresponding spatial periods are calculated and expressed in nanometers. This method allows quantifying the main periodic patterns present in the image. For control, one-dimensional (1D) FFT analysis was also performed, averaged line-by-line or row-by-row, after rotation correction, if necessary. By averaging the individual line-by-line or row-by-row 1D FFT measurements, phase shifts and outliers in the individual LIPSS were averaged out. The resulting spectrum was compared with the spectra of the 2D FFT for verification purposes. The results were very similar for both approaches, which means that the results obtained are highly likely to be meaningful. In cases where a rotation of the LIPSS could be observed, the image to be analyzed was rotated by a defined angle in Python using the SciPy image processing module. The exact value of the rotation angle was determined in advance using the directionality plug-in (Fourier components method) from ImageJ (v. 1.51).

### 2.4. Polymer Replicas

Titanium and steel samples were cleaned in 80% ethanol (Carl Roth GmbH + Co., KG, Karlsruhe, Germany) in a sonicator for 15 min. The air-dried samples were then covered with President Xtra lite (Coltene, Altstätten, CH, Switzerland) dental silicone and pushed into the silicone by hand, so that a rim of about 5 mm formed around the metal plates. The silicone was allowed to harden for 5 min before removing the metal plates.

Cellulose acetate-butyrate (CAB), which was provided by Sigma-Aldrich (Merck, Darmstadt, Germany), was dissolved in formic acid at a concentration of 15% (*w*/*v*) by slow overhead rotation in a 2 mL tube overnight. The solution was poured into the silicone molds and degassed briefly (2–5 s) in a desiccator, before allowing the formic acid to evaporate overnight in a fume hood. The next day, the CAB replicas were removed from the silicone molds and sterilized in an autoclave in phosphate-buffered saline (PBS) provided by Sigma-Aldrich, Wien, Austria.

### 2.5. Surface Profiles

Atomic force microscope (AFM) images were measured with a device from Nanosurf, Liestal, Switzerland. The images were taken in contact mode. Tilts in the images were compensated by AFM image processing software, which was also used to obtain the AFM profiles perpendicular to the LIPSS directions.

### 2.6. Cell Cultivation

In this study, murine Schwann cells (Immortalized Mouse Schwann Cells, IMS32; T0295, Applied Biological Materials Inc., Richmond, BC, Canada) were employed. They were cultured in 4 mL of growth medium comprising PriGrow III (TM003, Applied Biological Materials Inc., Richmond, BC, Canada) supplemented with 10% fetal bovine serum (FBS) and 1% Pen/Strep solution. For control experiments, murine fibroblasts (LTK cell line; ECACC, UK; catalog no. 85011425) were used. These cells were maintained in DMEM (Lactan, Graz, Austria) supplemented with 2 mM glutamine (Sigma-Aldrich; Merck, Darmstadt, Germany), 10% FBS (Biochrom, Berlin, Germany), and 50 U/mL penicillin plus streptomycin (Serva, Heidelberg, Germany). Before cell seeding, the samples were autoclaved for 1 h in PBS at 100 °C and 0.1 MPa. The seeding density of the cells was 25,000 cells/cm^2^. All cells were incubated at constant temperature of 37 °C with 5% CO_2_ and were passaged weekly at a 1:10 split ratio.

### 2.7. Preparation for Electron Microscope Investigation

After an incubation period of 10 days, the samples were collected and prepared for scanning electron microscopy. Following a PBS rinse, they were fixed overnight in 6.25% glutaraldehyde (SERVA Electrophoresis GmbH, Heidelberg, Germany) in PBS and dehydrated using a graded ethanol series. Final drying was performed overnight with HMDS (hexamethyldisilazane; Carl Roth GmbH + Co., KG, Karlsruhe, Germany) to minimize deformation and shrinkage artifacts. The dried samples were sputter-coated with gold for 80 s at 22 mA (sputter coater BAL-TEC SCD 005, Leica Microsystems, Wetzlar, Germany) and subsequently examined using an electron microscope SEM 525 M (Philips, Eindhoven, The Netherlands).

## 3. Results

### 3.1. Tests on Titanium Alloy Samples

**Figure 3:**
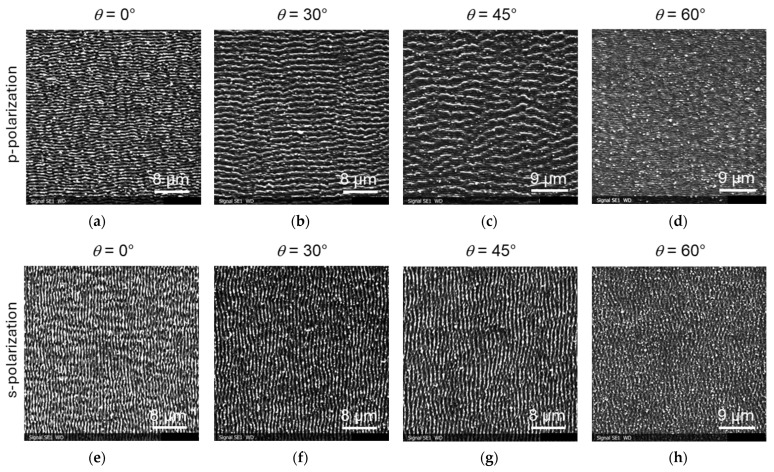
SEM images of LIPSS on inclined titanium alloy samples with *F*_0_ = 0.82 J/cm^2^: (**a**–**d**) irradiation with p-polarized light; (**e**–**h**) irradiation with s-polarized light.

In this section, we combine results of irradiation with s- and p-polarized light using the same range of parameters (i.e., fluences and angles). The LIPSS periodicity was determined using FFT transform. The surfaces were irradiated at a repetition rate, *f*, of 0.5 kHz, using a scan speed of 3000 µm/s and a scan spacing of 6 µm. This corresponds to a number of pulses per area, *N*_eff2D_, of 87. In the fluence range, *F*_0_, between 0.3 and 1.0 J/cm^2^, LIPSS were formed with a varying periodicity depending on the inclination angle. The angle dependence is similar for all fluences in this range. Figure 3 shows exemplary SEM results for different inclination angles, *θ*, at a fluence, *F*_0_, of 0.82 J/cm^2^.

**Figure 4:**
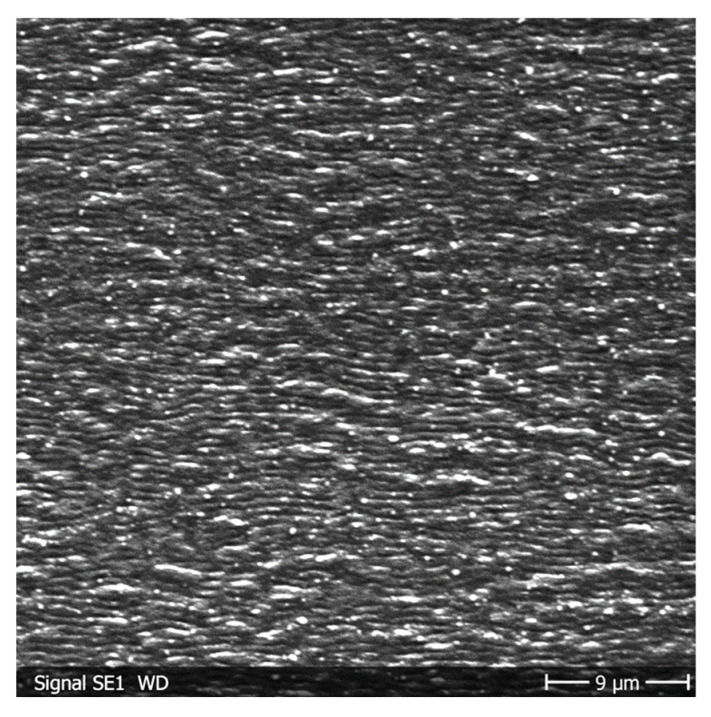
SEM image of overlapped smaller- and larger-size p- and p* LIPSS on titanium alloy samples inclined under *θ* = 45° at *F*_0_ = 0.5 J/cm^2^.

For p-polarization, a second larger-size LIPSS feature is observed at medium angles for all fluences, *F*_0_, between 0.3 and 1.0 J/cm^2^, as is shown in Figure 4 for a fluence of *F*_0_ = 0.5 J/cm^2^. This second LIPSS structure, which we address as p* structure, overlaps with the primary smaller p LIPSS structure and is in many cases dominant. The occurrence of the two different LIPSS features is especially obvious in the Fourier profiles, as shown in Figure 2c.

**Figure 5:**
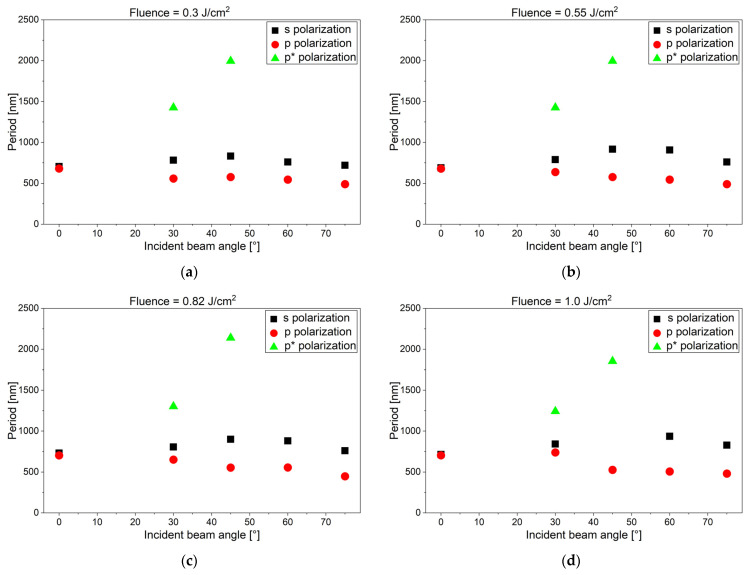
Angle dependence of LIPSS features on inclined titanium samples with a fluence, *F*_0_, of (**a**) 0.3 J/cm^2^, (**b**) 0.55 J/cm^2^, (**c**) 0.82 J/cm^2^, and (**d**) 1.0 J/cm^2^, respectively.

Figure 5 summarizes the results of LIPSS for different polarizations, inclination angles, and laser fluences (between 0.3 and 1.0 J/cm^2^). The irradiation of inclined samples allowed a variation in the periodicity of the LIPSS in a range between 450 and 900 nm. For p-polarization, a second larger-size p* LIPSS feature with periodicities between 1250 and 2150 nm is observed at medium angles.

Below *F*_0_ = 0.3 J/cm^2^, no LIPSS could be generated. In the fluence range between *F*_0_ = 1.0 J/cm^2^ and *F*_0_ = 2.0 J/cm^2^, s-polarization-induced LIPSS were only observed on flat samples, while p-polarization-induced LIPSS were observed also on tilted samples. In the latter case, the larger p* features were also observed especially for inclination angles of 30° and 45°.

### 3.2. Tests on Steel Samples

**Figure 6:**
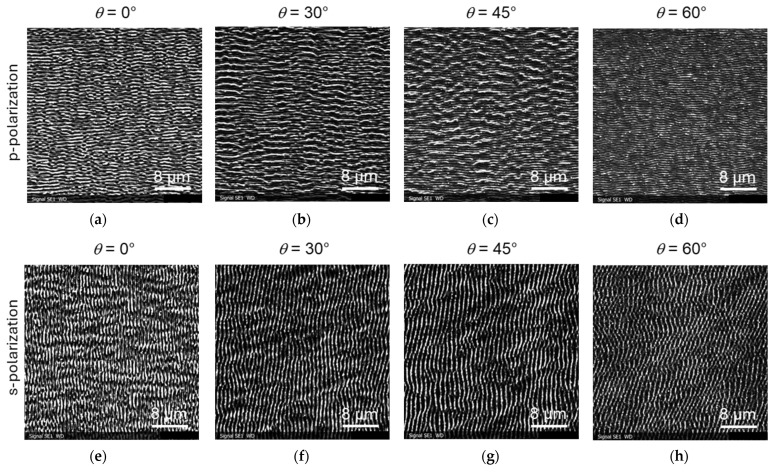
SEM images of LIPSS on inclined steel samples with *F*_0_ = 0.82 J/cm^2^: (**a**–**d**) irradiation with p-polarized light; (**e**–**h**) irradiation with s-polarized light.

**Figure 7:**
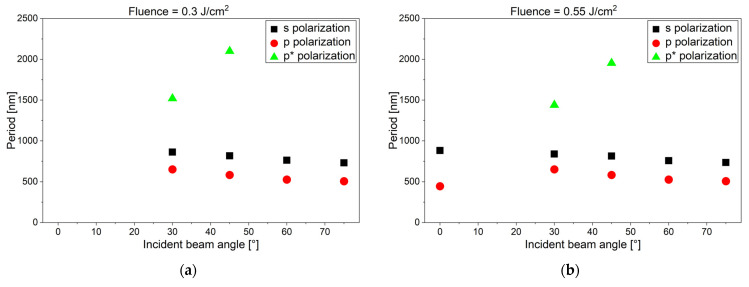

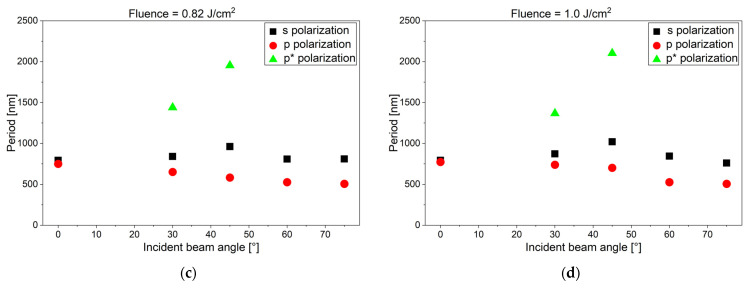
Angle dependence of LIPSS features on inclined steel samples with a fluence, *F*_0_, of (**a**) 0.3 J/cm^2^, (**b**) 0.55 J/cm^2^, (**c**) 0.82 J/cm^2^, and (**d**) 1.0 J/cm^2^, respectively.

Also for the steel samples, LIPSS were formed with a varying periodicity depending on the inclination angle in a fluence range, *F*_0_, between 0.3 and 1.0 J/cm^2^. The angle dependence is again similar for all fluences in this range. Figure 6 shows exemplary LIPSS induced by p- and s-polarized laser light on flat and tilted polished steel samples for *F*_0_ = 0.82 mJ/cm^2^. Similarly to titanium, we observed for p-polarization a second larger p* feature, especially for inclination angles of 30° and 45°. Figure 7 summarizes the results of LIPSS for different polarizations, inclination angles, and laser fluences (between 0.3 and 1.0 J/cm^2^). The irradiation of inclined steel samples allowed a variation in the periodicity of the LIPSS, in a range between 450 and 1050 nm. The second larger-size p* LIPSS feature varied between periodicities of 1450 and 2100 nm. These values are similar to those for titanium. Also, here, no LIPSS were observed for fluences *F*_0_ below 0.3 J/cm^2^. For steel, LIPSS occurred for both p- and s-polarized light on flat and tilted samples in the fluence range between *F*_0_ = 1.0 J/cm^2^ and *F*_0_ = 2.0 J/cm^2^. The larger p* features were present for inclination angles of 30° and 45°.

**Figure 8:**
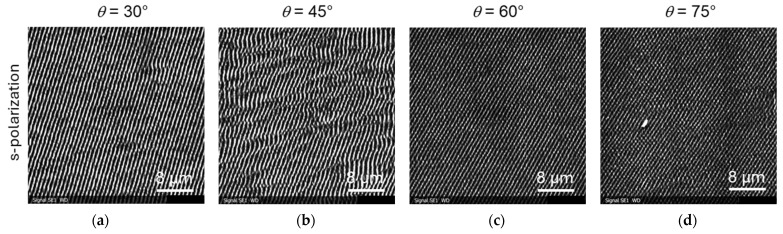
SEM images of LIPSS induced by s-polarized laser light on inclined steel samples with *F*_0_ = 0.55 J/cm^2^: (**a**–**d**) irradiation with s-polarized light.

**Figure 9:**
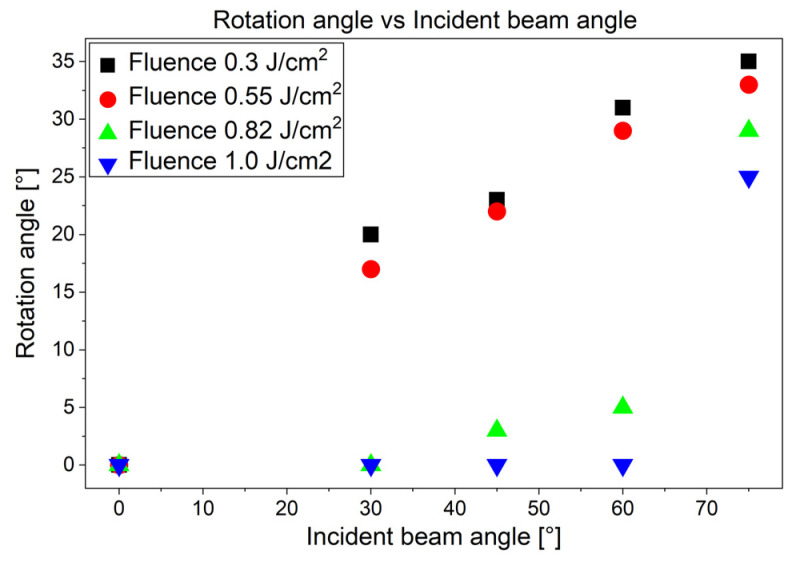
Structure rotation of LIPSS features for tilted surfaces for s-polarization-irradiated tilted steel samples in the fluence range of *F*_0_ = 0.3 to 1.0 J/cm^2^.

As can be seen in the images of Figure 6f–h, a certain rotation was observed for LIPSS induced by s-polarized light. This means that the ridges of the LIPSS are no longer strictly perpendicular to direction of the polarization of light, but have a certain rotation angle relative to the normal. This rotation increases with the tilt angle of the samples (or the angle of the incident beam). This effect was more pronounced for lower laser fluences, as is shown in Figure 8 for *F*_0_ = 0.55 mJ/cm^2^ and in the overview in Figure 9.

### 3.3. Comparison of Surface Profiles of Metal Masters and CAB Replicas

We could successfully replicate LIPSS with a range of different periodicities from titanium and steel samples into CAB. Assessment via SEM revealed that all fields of the stamps, like those shown in Figure 1b, were reproduced with unchanged periodicity.

**Figure 10:**
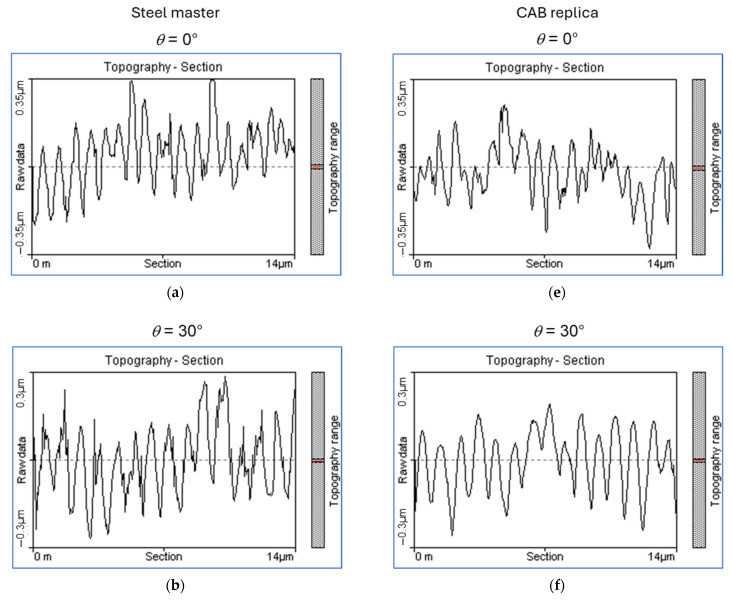

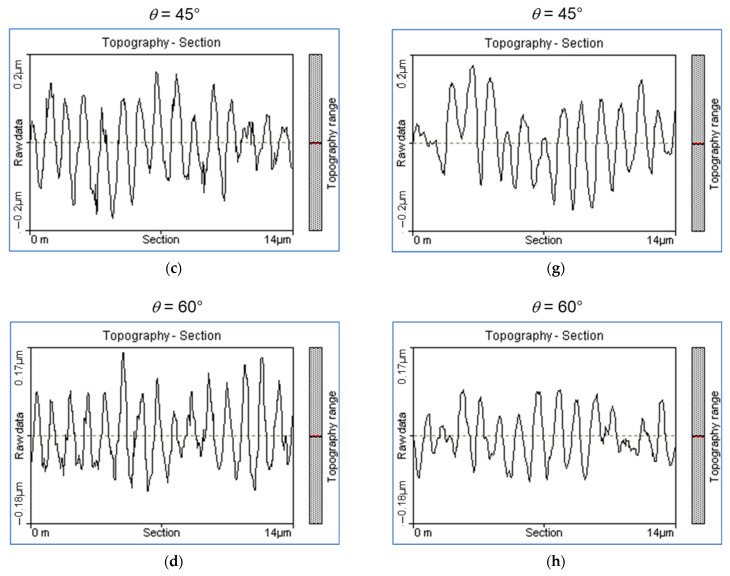
(**a**–**d**) AFM profiles of LIPSS on steel samples irradiated with s-polarized light of *F*_0_ = 0.82 J/cm^2^ under an angle of 0°, 30°, 45°, or 60°, respectively, and (**e**–**h**) AFM profiles from the corresponding CAB replicas taken from these areas. The horizontal scale of the profiles is 0 µm to 14 µm and vertical height scale ranges from −0.35 µm to 0.35 µm in (**a**,**e**), from −0.3 µm to 0.3 µm in (**b**,**f**), from −0.2 µm to 0.2 µm in (**c**,**g**), and from −0.18 µm to 0.17 µm in (**d**,**h**).

Figure 10 shows a comparison of AFM profiles of s-LIPSS on steel samples irradiated under different inclination angles with AFM profiles of the corresponding CAB replicas. The AFM images from which those profiles were derived are shown in Figure S1 in the Supplementary Materials. It is obvious that the structure heights of these features decrease with increasing inclination angle, even though the periodicity does not change much. Less pronounced is the effect that, at the same inclination angle, the CAB replicas seem to be slightly shallower than the corresponding LIPSS on the steel masters. But overall, the structure height is well reproduced after the two-step replication process.

### 3.4. Cell Attachment Tests on CAB Replicas

**Figure 11:**
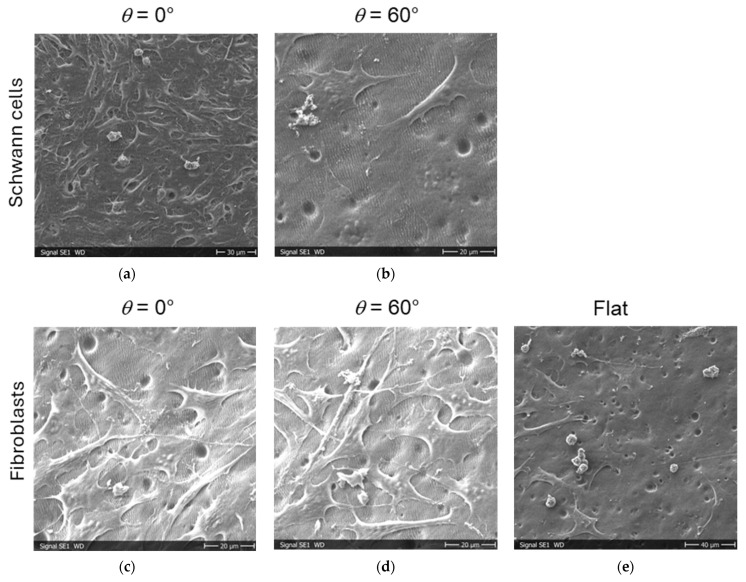
SEM images of cell attachment on CAB replicas on areas with LIPSS, generated with inclination angle of 0° and 60°, and flat control: (**a**,**b**) Schwann cells; (**c**–**e**) fibroblasts.

Schwann cells were seeded onto CAB replicas of titanium and steel samples with areas with LIPSS with different irradiation parameters. Later, the cells were fixed and investigated by SEM. The cells grew on the CAB surfaces, but we could observe no alignment of Schwann cells for all polarizations, inclination angles, and laser fluences under investigation. For control, the cell attachment experiments were repeated with fibroblasts with the identical qualitative result that fibroblasts also showed no alignment. Figure 11 shows results of the SEM investigation for CAB replicas of titanium and steel samples (for the experiments with Schwann cells and fibroblasts, respectively) with LIPSS generated by s-polarized laser light. The cells attach well to the surface with pronounced cell protrusions, e.g., the lamellipodia and the finer filopodia, but show no spindle shape and no orientation in the direction of the underlying LIPSS. In the area between the cells, the LIPSS are clearly visible, if one zooms into the images of Figure 11b,d. The spherical features in the images, most pronounced in the image of Figure 11e, are probably dead or dying cells that lost contact with the surface. It is also visible in the images of Figure 11 that the replicated CAB surface is somehow wavy on a scale of a few 10 µm and that there are (hemi-spherical) craters, which can be attributed to air bubbles generated during the replication process.

## 4. Discussion

We repeated the LIPSS generation experiments on titanium and steel samples independently in three experimental series, twice with the setup in Linz and a third time with another fs laser at Manutech-USD in St. Etienne. We could qualitatively reproduce most of the results reported above. Interestingly, some other publications reported on LIPSS formation on tilted steel and titanium samples and other materials using fs and other lasers; however, there was some discrepancy with the results of this work. In the Supplementary Materials, we have included a comparison of the key experimental parameters of our work with those of refs. [14,15,16,17,18,19] in Table S3. In refs. [15,16], an s-polarized fs fiber laser (wavelength of 1030 nm, pulse width 420 fs, repetition rate 100 kHz, high scanning speeds (compared to our work)) was used for LIPSS formation on tilted samples consisting of stainless steel (SUS 304, which is identical to our alloy 1.4301), titanium (not specified), or other materials. For both steel and titanium, the periodicity decreased with increasing tilt angle, while in our work the periodicity mostly first increased and later decreased again (see Figure 5 and Figure 7c,d). They also reported a rotation of the LIPSS orientation for both materials, while we see this effect only for steel and not for titanium. In ref. [17], a p-polarized fs laser (wavelength of 1030 nm, pulse width 222 fs, repetition rate 50 kHz, scanning speeds 200 mm/s) was used for LIPSS formation on tilted samples consisting of stainless steel (X5CrNi18-10, which is again identical to our alloy 1.4301) and silicon. A rotation of the LIPSS was reported for p-polarized light, while we saw this effect only for s-polarization. These discrepancies probably highlight the strong influence of experimental conditions (material, surface preparation, fluence range, and beam incidence geometry) on LIPSS formation and underline the complexity of predicting structure morphology. In particular, a possible directionality of the surface roughness was reported to have a pronounced influence on the LIPSS orientation [25,26]; however, this was for considerably rougher samples than in our work. This was, however, not the case for all samples in the literature [14,15,16,17], where polishing procedures were not done or at least not described.

**Figure 12:**
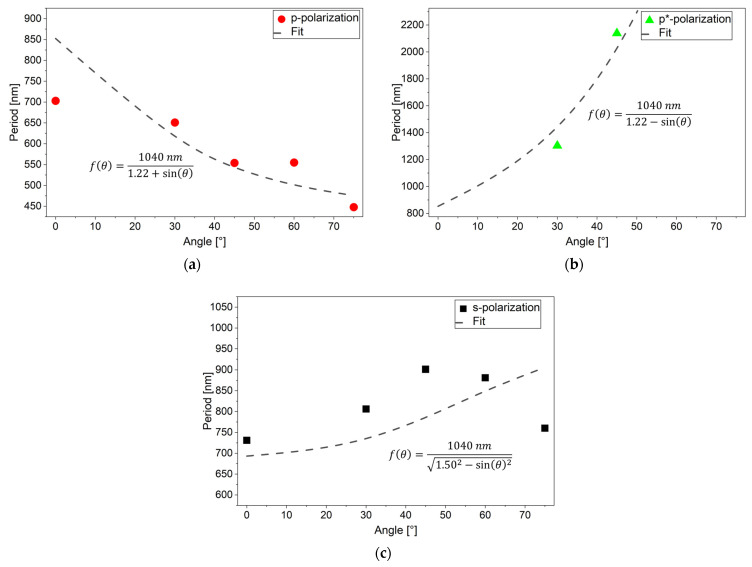
Fit of formulas from D. Bäuerle [27] to experimental data of LIPSS on tilted titanium samples for *F*_0_ = 0.82 J/cm^2^: (**a**) p features; (**b**) p* features, and (**c**) s features.

Another point that is not often addressed in the literature is the appearance of the larger p* features. But it is mentioned, for the materials of interest here, in [16] and discussed in detail in [19]. Both features, in our work called p and p*, are attributed to interference of the incoming laser light with surface plasmon polaritons (SPPs) running in opposite directions parallel to the p-polarization. In a book by D. Bäuerle [27], formulas of the angle dependence of p and p* features are derived based on simple geometrical considerations. There, a formula for the s features is also given, at least for LIPSS without rotation. Figure 12 shows the comparison between experimental results with fits to these formulas, where the effective index of refraction of the SPPs, *n*_SPP_ = 1.22 and 1.50, was used as fit parameter, using a least mean squares approach (to minimize the sum of the squares of the differences between observed and predicted values). For the p and p* features, a more sophisticated model, i.e., finite-difference time-domain (FDTD) simulations or comparable theoretical calculation diagrams, taking also the effects of the surface roughness into account [19], would be necessary for a deeper understanding and may lead to a better agreement, but the general trend is already reproduced. For the s features, the decrease in the period at the highest inclination angles cannot be explained with the fitted formula.

As for instance outlined in [19], the absorbed fraction of the laser pulse energy is also angle-dependent due to the corresponding dependence of the Fresnel reflection coefficient. So far, we have not taken this effect into account for our fluence calculations and just increased the fluence for inclined samples by a factor of 1/cos*θ*. The more complex angle dependence for s-polarization, with minimal reflection at the Bragg angle, may however explain the systematic discrepancy of the measured data to the fit in Figure 12c only in part. Another aspect is that the formula used for the fit in Figure 12c is only valid if the direction of the excited surface plasmon is strictly parallel to the s-polarization, which is probably not the case for rotated LIPSS on steel samples. Here, modified mathematical dependencies apply.

In some of the FFT analysis images, we additionally see normal LSF (low-spatial-frequency) LIPSS peaks, as well as peaks with considerably higher spatial frequencies. One interpretation is that these are HSF (high-spatial-frequency) LIPSS features, as described for instance in [7]. Another explanation would be the occurrence of higher harmonics in the FFT analysis, due to image processing artifacts. Indeed, in nearly all cases where we see these features, they have about half of the spatial frequency of a dominant feature and cannot been identified directly by eye in the original SEM image. In Figure 5 and Figure 7, therefore, we only show features which we could identify by eye in SEM images and which follow the trends of the neighboring data points.

**Figure 13:**
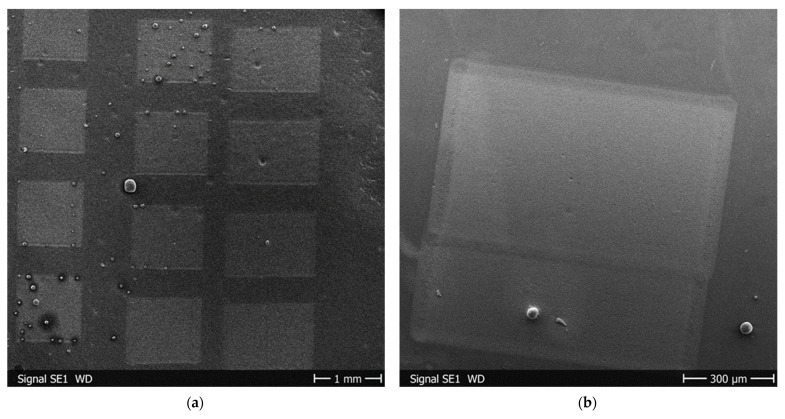

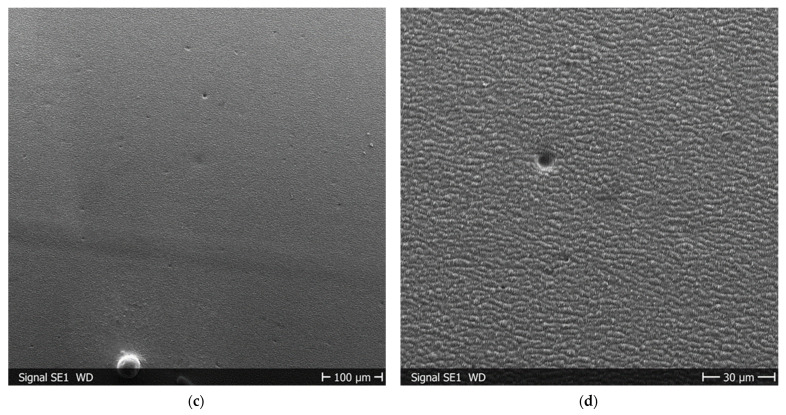
SEM images of distortions on CAB replicas of metal masters with LIPSS: (**a**) “Overview” image over several fields; (**b**) one field from another sample in (**a**); (**c**,**d**) magnified details of (**b**).

It was not possible to align either the relevant Schwann cells along the LIPSS on CAB or fibroblasts, which we investigated as a control. In our previous study, we demonstrated, however, that Schwann cells can indeed be aligned by growing them on polyethylene terephthalate (PET) foils with LIPSS with a periodicity of about 330 nm [28]. The cells there showed a pronounced spindle shape and the orientation was within a few degrees parallel to the direction of the LIPSS. Similar results were obtained for other cells and LIPSS on other polymers, if the structure periodicity was not too small [29]. We have no direct explanation for the discrepancy between the results here, shown in Figure 11, and the previous observations. One possibility could be the use of CAB, which may not be favorable for cell alignment via LIPSS due to its chemical composition or some ingredients. Another aspect is that the replication resulted in slightly shallower ripple structures than those on the titanium/steel masters, which may not provide enough stimuli for further cell alignment. Additionally, we observed some waviness on the scale of around 30 µm on the surface of the replicated CAB surface, as well as hemi-spherical craters with diameters of a few µm. Both features are visible also in the low-magnification SEM images of CAB replicas in Figure 13. Additionally, we see (especially in Figure 13a–c) bright “mushroom”-shaped protrusions, which are probably due to air bubbles in the dental silicone in the first replication step. Smaller, but with probably higher number density, are the dark “craters”, which are probably due to air bubbles in the CAB solution in the second replication step (visible especially in Figure 13b,c and also in Figure 11). In Figure 13d, one can the wavy structure, which runs more or less perpendicular to the underlying smaller LIPSS features. The origin of the wavy structures is not clear, but we saw them pronounced for steel masters at higher laser fluences. The large and not-too-dense protrusions are probably no problem for the alignment of the considerably smaller cells, while the small craters and wavy micro-features may disturb or override the influence of the nm-scale LIPSS for the alignment of the cells.

**Figure 14:**
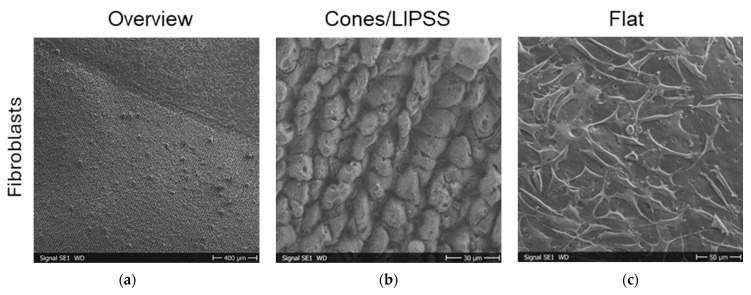
SEM images of cell attachment on CAB replicas of areas with fs-laser-induced cones (covered with LIPSS) and of flat control: (**a**) the “Overview” image shows the boundary between a laser-structured area (lower-left region) and the unstructured flat area (upper-right corner); (**b**) the image labeled “Cones/LIPSS” is a zoom-in of the lower-left region of the “Overview” image; (**c**) the image “Flat” is a zoom-in of the upper-right corner of the “Overview” image.

For even larger surface roughness replicated onto CAB, i.e., titanium masters with fs-laser-induced cones (or spikes) covered by LIPSS, we could reproduce the cell-repellent effect of the laser-structured titanium masters [24]. This latter result is shown in Figure 14. While the flat unstructured CAB surface was covered by fibroblasts, the areas with the replicated laser structure remained cell-free. For the replication, the same techniques were used as in the rest of the study.

Another aspect is that the laser radiation on the polymers leads not only to the formation of the surface structures, but also to chemical changes in the chemical composition, e.g., due to the dissociation of polymer molecules, leading to the activation of the surface. The change in the chemical surface composition is often accompanied by an increase in the hydrophilicity of the polymer surface. With the double replication of the surface structure, of course, no change in the polymer surface wettability occurs [30]. Corrugation of a hydrophilic surface can make it superhydrophilic, while a hydrophobic surface can become superhydrophobic (see for instance [31]). The latter is related to the effect of self-cleaning, which is well known as the Lotus effect and may explain the cell repulsion observed in Figure 14. An increase in the water contact angle due to surface roughness is also known for CAB materials [32]. We see a moderate increase in the water contact angle from 89° on flat areas of a CAB replica to 108° on CAB replicas of areas with fs-laser-induced cones covered with LIPSS (i.e., the roughest surface in this study). This is shown in Figure S2 in the Supplementary Materials. In our previous work [5], we have demonstrated that fs-laser-written lines with dimensions in the µm scale on CAB can induce alignment of Schwann cells. Whether this effect is due only to the structures or additionally requires activation via laser-induced changes in polymer surface chemistry is not clear. For this, one would have to perform further cell experiments on polymer replicas of lines written in metal masters, similarly to the LIPSS and laser-induced cones in the present work.

## 5. Conclusions

Laser-induced periodic surface structures (LIPSS) have the potential to induce an alignment of Schwann cells, a type of glial cells that can support the regeneration of nerve pathways by guiding the neuronal axons in the direction of their alignment. However, the direct formation of LIPSS on polymeric materials relevant for nerve regeneration conduits is challenging. This applies especially for the biocompatible and biodegradable polymer cellulose acetate butyrate (CAB). In this study, we generated variable LIPSS patterns by using different irradiation angles and polarization directions. We could successfully transfer the patterns from titanium and steel masters into CAB by a two-step replication process. We cultured Schwann cells on the nanostructured CAB, but observed no alignment of cells for all LIPSS periodicities under investigation, which ranged from about 500 and 2200 nm. However, we could demonstrate other structure-mediated effects on Schwann cells, especially cell repellence, on replicated laser-induced structures on CAB. An optimization of the replicated LIPSS regarding structure height and an improved overall surface flatness of the replicated surface may enable also cell-alignment on CAB in future studies.

## Data Availability

The original contributions presented in this study are included in the article/Supplementary Materials. Further inquiries can be directed to the corresponding author.

## Supplementary Materials

The following supporting information can be downloaded at https://www.mdpi.com/article/10.3390/ma19061091/s1. Table S1: Metal sample composition; Table S2: 2D FFT code; Table S3: Comparison of key experimental parameters; Figure S1: AFM images of laser-processed areas on steel master and corresponding CAB replica; Figure S2: Water contact angle measurements on flat and laser-processed CAB replicas.

## Abbreviations

The following abbreviations are used in this manuscript:

1D	One-dimensional
2D	Two-dimensional
AFM	Atomic force microscope
CAB	Cellulose acetate butyrate
ECM	Extracellular matrix
ELI	Extra Low Interstitial
FBS	Fetal bovine serum
FDTD	Finite-difference time-domain
FFT	Fast Fourier Transform
HMDS	Hexamethyldisilazane
HSF	High spatial frequency
LIPSS	Laser-induced periodic surface structures
LSF	Low spatial frequency
PBS	Phosphate-buffered saline
PET	Polyethylene terephthalate
PC	Polycarbonate
SEM	Scanning electron microscope
UK	United Kingdom
YAG	Yttrium Aluminum garnet
