# Supplementary material for: Polymer Replicas of Fs-Laser-Induced Periodic Surface Structures for Cell Attachment
Source: Materials (Basel). 2026 Mar 12;19(6):1091. doi: 10.3390/ma19061091 (PMC13028253; doi:10.3390/ma19061091)
Supplement: Supplementary file 1 [file materials-19-01091-s001.zip › Supporting Information1_JH110326.pdf]

Supporting Information 1

Polymer replicas of fs-laser-induced periodic surface structures for cell attachment

Prunella Ndjogo [1], Marion Widhalm [2,3], Agnes Weth [3], Sebastian Lifka [3], Werner Baumgartner [3], Yoan Di Maio [1] and Johannes Heitz [2,*]

1 Manutech-USD, 20 rue Pr. Benoit Lauras, 42000 St. Etienne, France; prunella.ndjogo@manutech-usd.fr (P.N.); yoan.di-maio@manutech-usd.fr (Y.D.M.)

2 Institute of Applied Physics, Johannes Kepler University Linz, Altenberger Strasse 69, 4040 Linz, Austria; marion.widhalm@jku.at (M.W.); johannes.heitz@jku.at (J.H.)

3 Institute of Biomedical Mechatronics, Johannes Kepler University Linz, Altenberger Strasse 69, 4040 Linz, Austria; marion.widhalm@jku.at (M.W.); agnes.weth@jku.at (A.W.); sebastian.lifka@jku.at (S.L.); werner.baumgartner@jku.at (W.B.)

* Correspondence: johannes.heitz@jku.at

Table S1. Metal samples composition.

**TiAl6V4 ELI**	**wt%**		**Material No. 1.4301**	**wt%**
Fe	≤ 0.25		C	≤ 0.07
C	≤ 0.08		Si	≤ 1.00
N	≤ 0.05		Mn	≤ 2.00
O	≤ 0.13		P	≤ 0.045
H	≤ 0.012		Si	≤ 0.015
Al	5.50 – 6.50		Cr	17.5-19.5
V	3.50 – 4.50		Ni	8.00-10.5
Ti	Rest		N	≤ 0.11
			Fe	Rest
