# Supplementary material for: Polymer Replicas of Fs-Laser-Induced Periodic Surface Structures for Cell Attachment
Source: Materials (Basel). 2026 Mar 12;19(6):1091. doi: 10.3390/ma19061091 (PMC13028253; doi:10.3390/ma19061091)
Supplement: Supplementary file 1 [file materials-19-01091-s001.zip › Supporting Information2_JH110326.pdf]

Supporting Information 2

Polymer replicas of fs-laser-induced periodic surface structures for cell attachment

Prunella Ndjogo [1], Marion Widhalm [2,3], Agnes Weth [3], Sebastian Lifka [3], Werner Baumgartner [3], Yoan Di Maio [1] and Johannes Heitz [2,*]

1 Manutech-USD, 20 rue Pr. Benoit Lauras, 42000 St. Etienne, France; prunella.ndjogo@manutech-usd.fr (P.N.); yoan.di-maio@manutech-usd.fr (Y.D.M.)

2 Institute of Applied Physics, Johannes Kepler University Linz, Altenberger Strasse 69, 4040 Linz, Austria; marion.widhalm@jku.at (M.W.); johannes.heitz@jku.at (J.H.)

3 Institute of Biomedical Mechatronics, Johannes Kepler University Linz, Altenberger Strasse 69, 4040 Linz, Austria; marion.widhalm@jku.at (M.W.); agnes.weth@jku.at (A.W.); sebastian.lifka@jku.at (S.L.); werner.baumgartner@jku.at (W.B.)

* Correspondence: johannes.heitz@jku.at

Table S2. 2D FFT code.

```
from PIL import Image

import numpy as np

import matplotlib.pyplot as plt

from scipy.ndimage import gaussian_filter

from scipy.signal import find_peaks

# --- Paramètres d'échelle ---

scale =25.79 # pixels par µm #9µm ->23.56 #8µm ->25.79 #4µm ->46.75 #10µm ->19 #6µm -
>35.77 #4µm ->46.25

step = 1 / scale

# --- Chargement de l'image ---

image_path = r"C:\Users\AK903459\Documents\Prunella\Stainless steel works\tests with

angles\s and p polarization images\P polarization\img45°/13mW.tif"

image = np.asanyarray(Image.open(image_path), dtype=float)

# --- Normalisation ---

image = (image - np.min(image)) / (np.max(image) - np.min(image))

# --- FFT 2D ---

F = np.fft.fft2(image)

Fshift = np.fft.fftshift(F)
```

```
magnitude = np.abs(Fshift)

# --- Coordonnées fréquentielles ---

ny, nx = image.shape

fx = np.fft.fftshift(np.fft.fftfreq(nx, d=step))

fy = np.fft.fftshift(np.fft.fftfreq(ny, d=step))

FX, FY = np.meshgrid(fx, fy)

FR = np.sqrt(FX**2 + FY**2) # fréquence radiale

# --- Masque pour ignorer basse fréquence ---

mask = FR > 0.05

magnitude_masked = magnitude * mask

# --- Flou léger pour réduire artefacts ---

magnitude_smooth = gaussian_filter(magnitude_masked, sigma=1.0)

# --- Profil radial (moyenne azimutale) ---

r_vals = np.linspace(0, FR.max(), 500)

radial_profile = np.zeros_like(r_vals)

for i, r in enumerate(r_vals):

    shell = (FR >= r - 0.5*(r_vals[1]-r_vals[0])) & (FR < r + 0.5*(r_vals[1]-r_vals[0]))

    if np.any(shell):

        radial_profile[i] = magnitude_smooth[shell].mean()

# --- Détection des pics ---

peaks, _ = find_peaks(radial_profile, distance=10) # "distance" évite les faux pics trop proches

# trier par amplitude décroissante et garder les deux plus forts

sorted_peaks = peaks[np.argsort(radial_profile[peaks])[::-1][:2]]

# --- Conversion en périodes ---

for idx in sorted_peaks:
```

```
        f = r_vals[idx]

        period_um = 1 / f

        period_nm = period_um * 1000

        print(f"Pic trouvé à f = {f:.3f} 1/µm -> période ≈ {period_nm:.1f} nm")

# --- Affichage ---

plt.figure(figsize=(18,5))


# 1) Image originale

plt.subplot(1,3,1)

plt.imshow(image, cmap="gray")

plt.title("SEM Image")

plt.axis("off")

# 2) Spectre FFT

plt.subplot(1,3,2)

plt.imshow(np.log1p(magnitude_smooth), cmap="inferno", extent=[fx.min(), fx.max(), fy.min(),
fy.max()])

plt.colorbar(label="log(|FFT|)")

plt.title("FFT spectre (log-scale)")

plt.xlabel("fx (1/µm)")

plt.ylabel("fy (1/µm)")

# 3) Profil radial

plt.subplot(1,3,3)

plt.plot(r_vals, radial_profile, label="Radial profile")

plt.plot(r_vals[sorted_peaks], radial_profile[sorted_peaks], "ro", markersize=8, label="Detected
peaks")
```

```
# Décalage pour les annotations
for idx in sorted_peaks:
    plt.text(r_vals[idx] + 0.05,      # décale légèrement en x
        radial_profile[idx] + 0.02*radial_profile.max(), # décale légèrement en y
        f"{1/r_vals[idx]*1000:.0f} nm",
        color="red", fontsize=9, ha="left")
plt.xlabel("Spatial frequency (1/µm)")
plt.ylabel("Average amplitude")
plt.title("FFT radial profile")
plt.legend()
plt.grid(True)
plt.tight_layout()
plt.show()
```
