# Supplementary material for: Polymer Replicas of Fs-Laser-Induced Periodic Surface Structures for Cell Attachment
Source: Materials (Basel). 2026 Mar 12;19(6):1091. doi: 10.3390/ma19061091 (PMC13028253; doi:10.3390/ma19061091)
Supplement: Supplementary file 1 [file materials-19-01091-s001.zip › Supporting Information3_JH110326.pdf]

Supporting Information 3

Polymer replicas of fs-laser-induced periodic surface structures for cell attachment

Prunella Ndjogo [1], Marion Widhalm [2,3], Agnes Weth [3], Sebastian Lifka [3], Werner Baumgartner [3], Yoan Di Maio [1] and Johannes Heitz [2,*]

1 Manutech-USD, 20 rue Pr. Benoit Lauras, 42000 St. Etienne, France; prunella.ndjogo@manutech-usd.fr (P.N.); yoan.di-maio@manutech-usd.fr (Y.D.M.)

2 Institute of Applied Physics, Johannes Kepler University Linz, Altenberger Strasse 69, 4040 Linz, Austria; marion.widhalm@jku.at (M.W.); johannes.heitz@jku.at (J.H.)

3 Institute of Biomedical Mechatronics, Johannes Kepler University Linz, Altenberger Strasse 69, 4040 Linz, Austria; marion.widhalm@jku.at (M.W.); agnes.weth@jku.at (A.W.); sebastian.lifka@jku.at (S.L.); werner.baumgartner@jku.at (W.B.)

* Correspondence: johannes.heitz@jku.at

Table S3. Comparison of key experimental parameters.

Source	Laser wave-length [nm]	Pulse width [fs]	Scanning speed [mm/s]	Repetition rate [kHz]	Spot size $2w_0$ [µm]	Pulse number per area	Fluence range [J/cm^2]	Materials under investigation
Current work	1040	350	3	0.5	62.4	87	0.3 - 2.0	Ti, steel
Isenor, 1977 [14]	9290, 10270, 10590	50 10^6	0	-	~ 100	5 -25	~ 5 - 25	Ni_xP_{1-x}
Liu et al., 2020 [15]	1030, 515	420	10 - 1500	100	60, 50	-	-	Ti, steel, Al, Cu
Liu et al., 2021 [16]	1030	420	150 - 300	100	60	-	-	steel
Schwarz et al. 2017 [17]	1030	222	200	50	31	5	5 - 8.5	steel, Si, SiO_2
Fuentes-Edfuf et al., 2019 [18]	800	100	-	0.1	-	50 - 1000	0.015 - 0.035	Cu
Fuentes-Edfuf et al., 2019 [19]	800	120	-	0.1	44, 59	20 - 200	1.0	steel
