# Supplementary material for: Polymer Replicas of Fs-Laser-Induced Periodic Surface Structures for Cell Attachment
Source: Materials (Basel). 2026 Mar 12;19(6):1091. doi: 10.3390/ma19061091 (PMC13028253; doi:10.3390/ma19061091)

Supporting Information 4

Polymer replicas of fs-laser-induced periodic surface structures for cell attachment

Prunella Ndjogo [1], Marion Widhalm [2,3], Agnes Weth [3], Sebastian Lifka [3], Werner Baumgartner [3], Yoan Di Maio [1] and Johannes Heitz [2,*]

1 Manutech-USD, 20 rue Pr. Benoit Lauras, 42000 St. Etienne, France; prunella.ndjogo@manutech-usd.fr (P.N.); yoan.di-maio@manutech-usd.fr (Y.D.M.)

2 Institute of Applied Physics, Johannes Kepler University Linz, Altenberger Strasse 69, 4040 Linz, Austria; marion.widhalm@jku.at (M.W.); johannes.heitz@jku.at (J.H.)

3 Institute of Biomedical Mechatronics, Johannes Kepler University Linz, Altenberger Strasse 69, 4040 Linz, Austria; marion.widhalm@jku.at (M.W.); agnes.weth@jku.at (A.W.); sebastian.lifka@jku.at (S.L.); werner.baumgartner@jku.at (W.B.)

* Correspondence: johannes.heitz@jku.at

Figure S1. AFM images of laser-processed areas on steel master and corresponding CAB replica.

Atomic force microscope (AFM) images were measured with a device from Nanosurf, Liestal, Switzerland. The images were taken in the contact mode. Tilts in the images were compensated by image processing software of the AFM, which is also used to obtain the AFM profiles perpendicular to the LIPSS directions.

Figure S1 (a). AFM image and profile of LIPSS on a steel sample irradiated with s-polarized light of F_0 = 0.82 J/cm^2 under an angle of 0°.

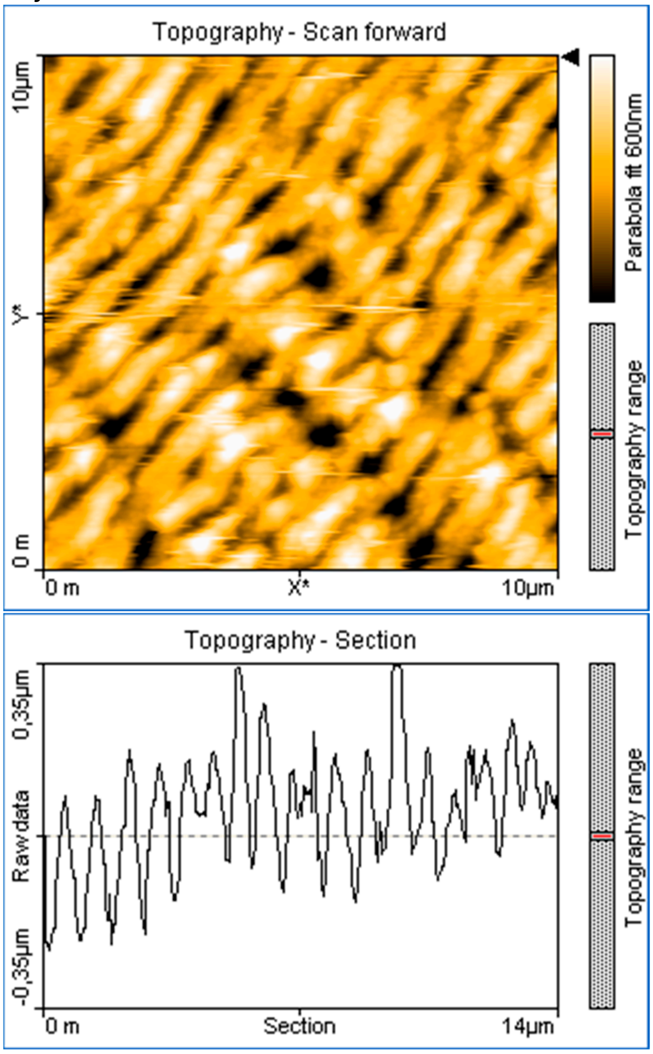

Figure S1 (b). AFM image and profile of a CAB replica of LIPSS on a steel sample irradiated with s-polarized light of F_0 = 0.82 J/cm^2 under an angle of 0°.

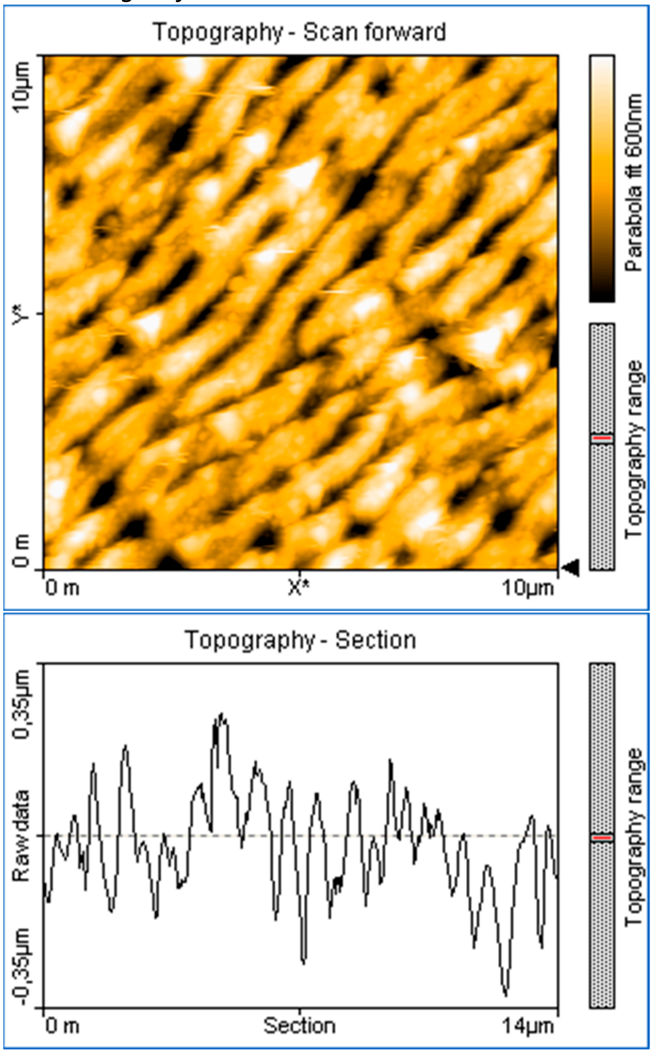

Figure S1 (c). AFM image and profile of LIPSS on a steel sample irradiated with s-polarized light of F_0 = 0.82 J/cm^2 under an angle of 30°.

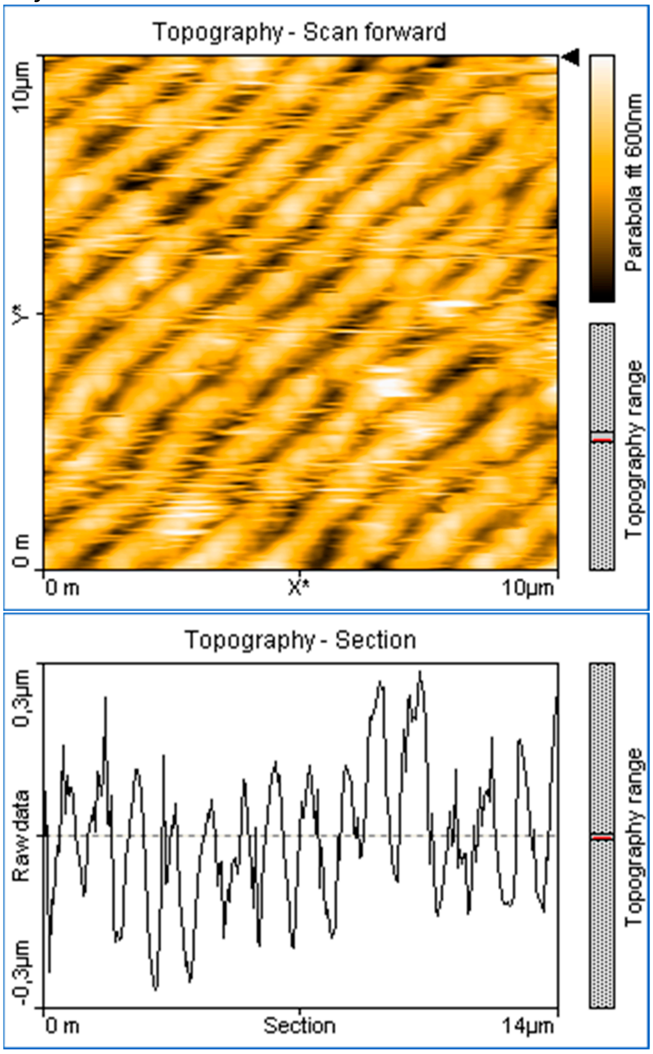

Figure S1 (d). AFM image and profile of a CAB replica of LIPSS on a steel sample irradiated with s-polarized light of F_0 = 0.82 J/cm^2 under an angle of 30°.

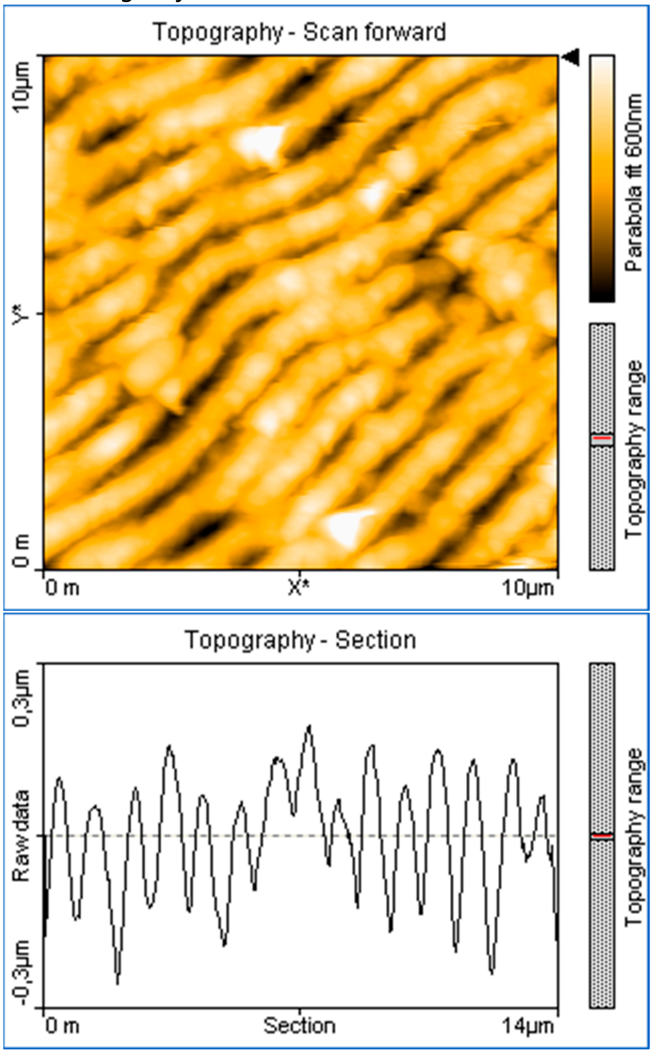

Figure S1 (e). AFM image and profile of LIPSS on a steel sample irradiated with s-polarized light of F_0 = 0.82 J/cm^2 under an angle of 45°.

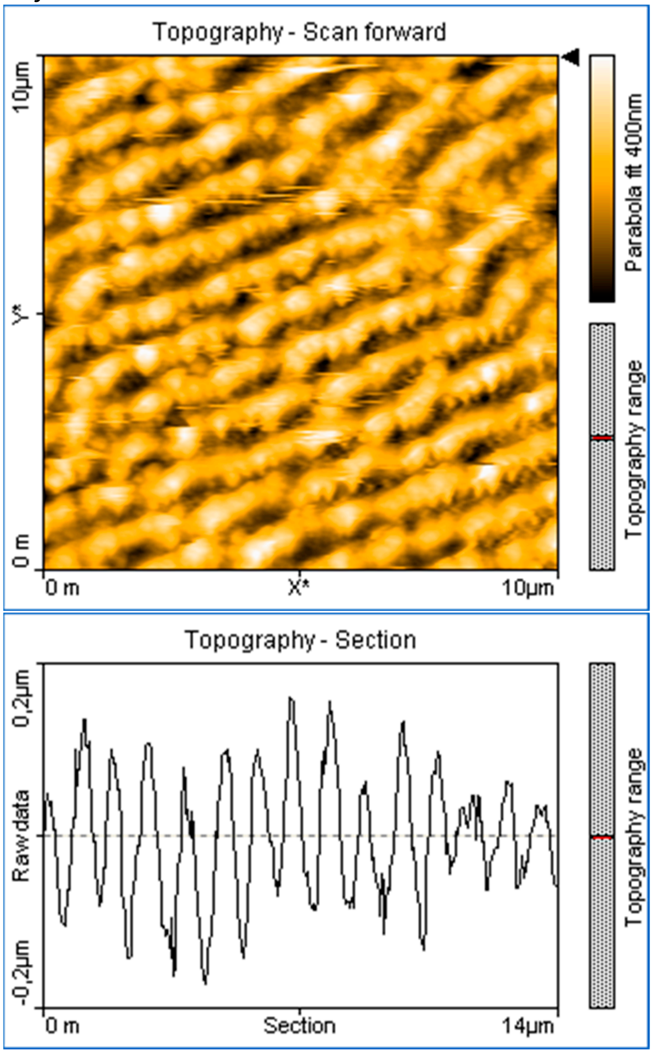

Figure S1 (f). AFM image and profile of a CAB replica of LIPSS on a steel sample irradiated with s-polarized light of F_0 = 0.82 J/cm^2 under an angle of 45°.

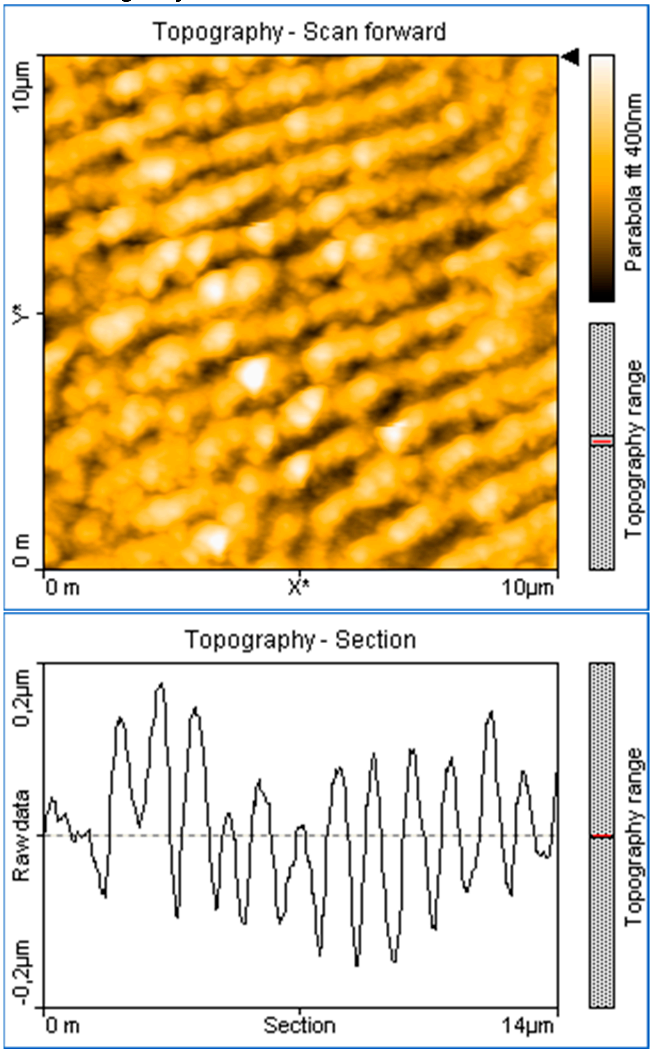

Figure S1 (g). AFM image and profile of LIPSS on a steel sample irradiated with s-polarized light of F_0 = 0.82 J/cm^2 under an angle of 60°.

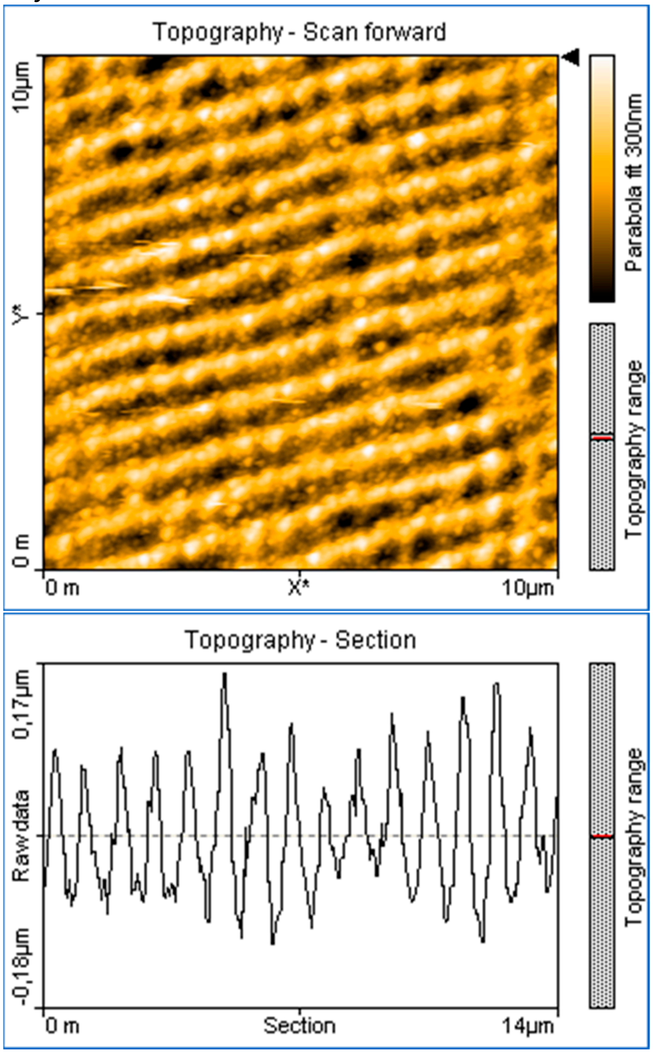

Figure S1 (h). AFM image and profile of a CAB replica of LIPSS on a steel sample irradiated with s-polarized light of F_0 = 0.82 J/cm^2 under an angle of 60°.

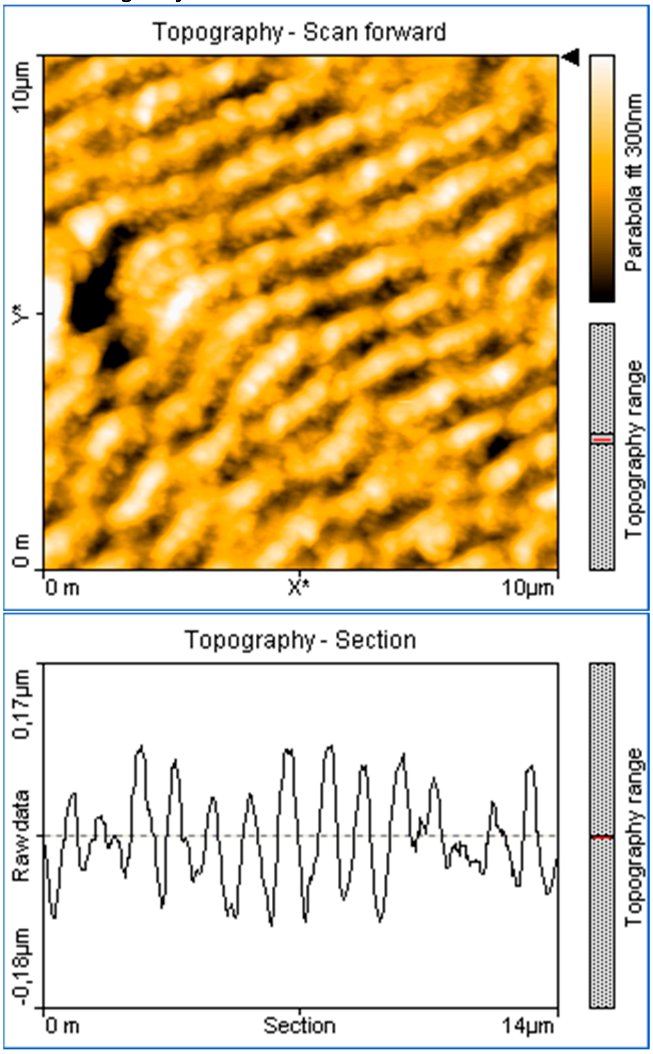

Supplement: Supplementary file 1 [file materials-19-01091-s001.zip › Supporting Information4_JH110326.pdf]
