# Supplementary material for: Polymer Replicas of Fs-Laser-Induced Periodic Surface Structures for Cell Attachment
Source: Materials (Basel). 2026 Mar 12;19(6):1091. doi: 10.3390/ma19061091 (PMC13028253; doi:10.3390/ma19061091)

Supporting Information 5

Polymer replicas of fs-laser-induced periodic surface structures for cell attachment

Prunella Ndjogo [1], Marion Widhalm [2,3], Agnes Weth [3], Sebastian Lifka [3], Werner Baumgartner [3], Yoan Di Maio [1] and Johannes Heitz [2,*]

[1] Manutech-USD, 20 rue Pr. Benoit Lauras, 42000 St. Etienne, France; prunella.ndjogo@manutech-usd.fr (P.N.); yoan.di-maio@manutech-usd.fr (Y.D.M.)

[2] Institute of Applied Physics, Johannes Kepler University Linz, Altenberger Strasse 69, 4040 Linz, Austria; marion.widhalm@jku.at (M.W.); johannes.heitz@jku.at (J.H.)

[3] Institute of Biomedical Mechatronics, Johannes Kepler University Linz, Altenberger Strasse 69, 4040 Linz, Austria; marion.widhalm@jku.at (M.W.); agnes.weth@jku.at (A.W.); sebastian.lifka@jku.at (S.L.); werner.baumgartner@jku.at (W.B.)

* Correspondence: johannes.heitz@jku.at

Figure S2. Water contact angle measurements on flat and laser-processed CAB replicas.

Static water contact angle measurements were performed in a home-build setup using the sessile drop method. The contact and surface lines were adjusted manually in the electronic image of the drop on the sample and then the angle of the contact line to the surface was determined.

Figure S2. Water contact angle measurements on CAB replicas of areas with fs-laser induced cones covered with LIPSS (left column) and of flat controls (right column).

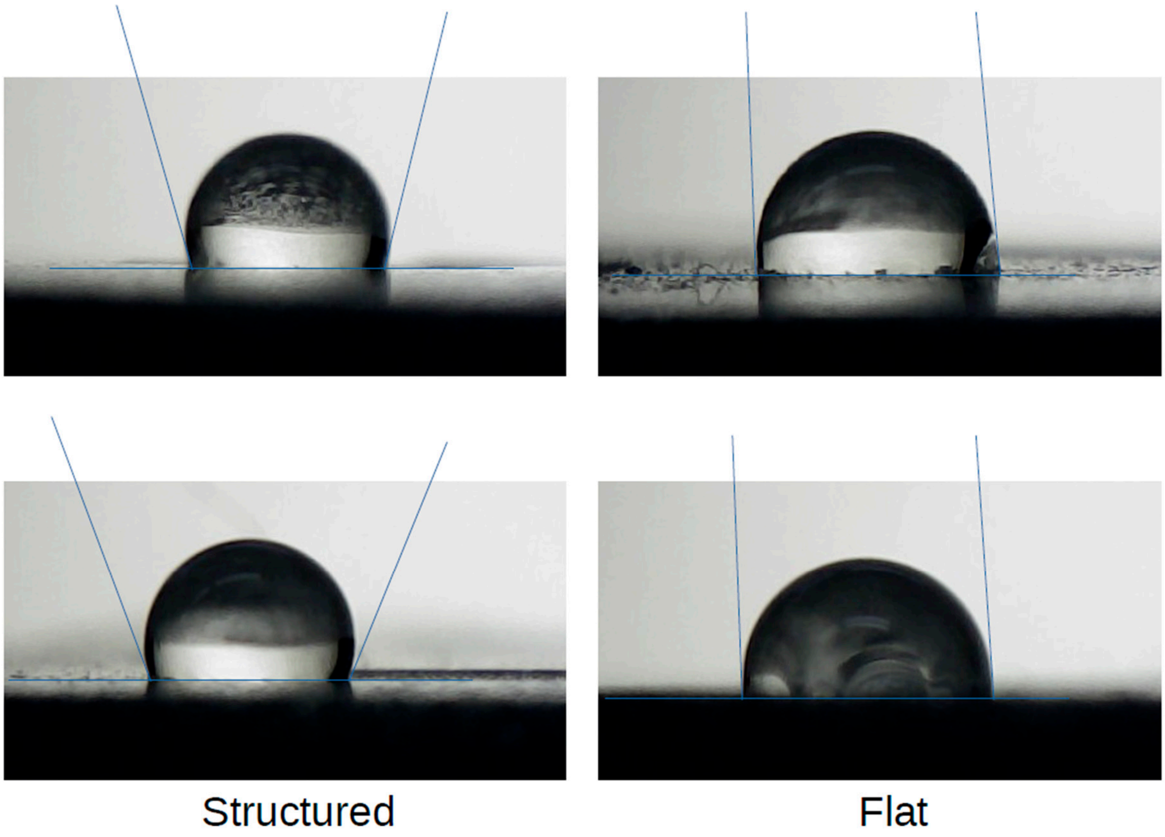

Supplement: Supplementary file 1 [file materials-19-01091-s001.zip › Supporting Information5_JH110326.pdf]
